# Interventions to improve adherence to treatment for paediatric tuberculosis in low- and middle-income countries: a systematic review and meta-analysis

**DOI:** 10.2471/BLT.14.147231

**Published:** 2015-06-23

**Authors:** Meaghann S Weaver, Knut Lönnroth, Scott C Howard, Debra L Roter, Catherine G Lam

**Affiliations:** a St Jude Children’s Research Hospital, 262 Danny Thomas Place, MS 721, Memphis, TN 38105, United States of America (USA).; b Global Tuberculosis Programme, World Health Organization, Geneva, Switzerland.; c World Child Cancer USA, Denver, USA.; d Johns Hopkins Bloomberg School of Public Health, Baltimore, USA.

## Abstract

**Objective:**

To assess the design, delivery and outcomes of interventions to improve adherence to treatment for paediatric tuberculosis in low- and middle-income countries and develop a contextual framework for such interventions.

**Methods:**

We searched PubMed and Cochrane databases for reports published between 1 January 2003 and 1 December 2013 on interventions to improve adherence to treatment for tuberculosis that included patients younger than 20 years who lived in a low- or middle-income country. For potentially relevant articles that lacked paediatric outcomes, we contacted the authors of the studies. We assessed heterogeneity and risk of bias. To evaluate treatment success – i.e. the combination of treatment completion and cure – we performed random-effects meta-analysis. We identified areas of need for improved intervention practices.

**Findings:**

We included 15 studies in 11 countries for the qualitative analysis and of these studies, 11 qualified for the meta-analysis – representing 1279 children. Of the interventions described in the 15 studies, two focused on education, one on psychosocial support, seven on care delivery, four on health systems and one on financial provisions. The children in intervention arms had higher rates of treatment success, compared with those in control groups (odds ratio: 3.02; 95% confidence interval: 2.19–4.15). Using the results of our analyses, we developed a framework around factors that promoted or threatened treatment completion.

**Conclusion:**

Various interventions to improve adherence to treatment for paediatric tuberculosis appear both feasible and effective in low- and middle-income countries.

## Résumé

**Objectif:**

Évaluer la conception, la mise en œuvre et les résultats des interventions visant à améliorer l'observance thérapeutique dans le traitement de la tuberculose chez l'enfant dans les pays à revenu faible et intermédiaire et élaborer un cadre contextuel pour ce type d'interventions.

**Méthodes:**

Nous avons fait des recherches dans les bases de données PubMed et Cochrane pour trouver des rapports publiés entre le 1er janvier 2003 et le 1er décembre 2013 sur des interventions visant à améliorer l'observance thérapeutique dans le traitement de la tuberculose chez des patients de moins de vingt ans vivant dans des pays à revenu faible ou intermédiaire. Pour les articles potentiellement pertinents qui omettaient de citer spécifiquement les résultats chez l'enfant, nous avons contacté les auteurs. Nous avons évalué l'hétérogénéité et le risque de biais. Pour évaluer la réussite d'un traitement (c'est-à-dire: combinaison de l'achèvement du traitement et de la guérison), nous avons effectué une méta-analyse à effets aléatoires. Nous avons également identifié les points à améliorer en vue d’optimiser les programmes d'intervention.

**Résultats:**

Pour notre analyse qualitative, nous avons intégré quinze études, menées dans onze pays. Sur ces études, onze ont pu être retenues pour la méta-analyse (représentant 1.279 enfants). Concernant les interventions décrites dans les quinze études, deux ciblaient l'éducation, une le soutien psychosocial, sept la délivrance des soins, quatre les systèmes de santé et une le soutien financier. Des taux de succès du traitement plus élevés ont été constatés chez les enfants qui ont bénéficié des interventions, comparativement aux enfants des groupes témoins (rapport des cotes: 3,02; intervalle de confiance de 95%: 2,19-4,15). À partir des résultats de nos analyses, nous avons conçu un cadre autour des facteurs ayant favorisé ou entravé l'achèvement du traitement.

**Conclusion:**

Plusieurs interventions visant à améliorer l'observance thérapeutique dans le traitement de la tuberculose chez l'enfant semblent être à la fois réalisables et efficaces dans les pays à revenu faible et intermédiaire.

## Resumen

**Objetivo:**

Evaluar el diseño, la prestación y los resultados de las intervenciones para mejorar el cumplimiento del tratamiento de la tuberculosis pediátrica en países de ingresos bajos y medios y desarrollar un marco contextual para tales intervenciones.

**Métodos:**

Se realizaron búsquedas en las bases de datos PubMed y Cochrane para encontrar informes sobre las intervenciones para mejorar el cumplimiento del tratamiento de la tuberculosis publicados entre el 1 de enero de 2003 y el 1 de diciembre de 2013 que incluyeran pacientes menores de 20 años que vivieran en países de ingresos bajos o medios. Se contactó con los autores de los estudios con artículos relevantes que carecían de resultados pediátricos. Se evaluó la heterogeneidad y el riesgo de sesgo. Se llevaron a cabo metanálisis de efectos aleatorios para evaluar el éxito del tratamiento, es decir, la combinación de finalización del tratamiento y cura. Se identificaron áreas que necesitaban una mejora de las prácticas de intervención.

**Resultados:**

Se incluyeron 15 estudios en 11 países para el análisis cualitativo y, de esos estudios, 11 cumplieron los requisitos para el metanálisis: una representación de 1279 niños. De las intervenciones descritas en los 15 estudios, dos se centraban en la educación, uno en el apoyo psicosocial, siete en la prestación de asistencia, cuatro en los sistemas de salud y uno en las dotaciones financieras. Los niños en el brazo de intervención tenían una tasa mayor de éxito del tratamiento en comparación con aquellos en grupos de control (razón de posibilidades: 3,02; intervalo de confianza del 95%: 2.19–4.15). Utilizando los resultados en los análisis, se desarrolló un marco alrededor de los factores que promovían o amenazaban la finalización del tratamiento.

**Conclusión:**

Varias intervenciones para mejorar el cumplimiento del tratamiento de la tuberculosis pediátrica parecen tanto viables como efectivas en países de ingresos bajos y medios.

## ملخص

الغرض تقييم التصميم والتقديم والنتائج المترتبة على عمليات التدخل بغرض رفع مستوى الالتزام بالعلاج المقدم لحالات السلّ لدى الأطفال في البلدان محدودة ومتوسطة الدخل ووضع إطار سياقي لعمليات التدخل من هذا النوع.

**الطريقة:**

لقد أجرينا بحثًا في قواعد بيانات PubMed وCochrane لإيجاد التقارير المنشورة في الفترة ما بين أول يناير/كانون الثاني من عام 2003 وأول ديسمبر/كانون الأول من عام 2013 حول عمليات التدخل الساعية إلى تحسين مستوى الالتزام بالعلاج المقدم لمرض السلّ والتي تضمنت مرضى تقل أعمارهم عن 20 عامًا ممن كانوا يعيشون في بلدان محدودة أو منخفضة الدخل. ولكي يتم إيجاد المقالات التي يحتمل أن تكون ذات صلة لكن ينقصها المحصلات المرتبطة بالمرضى من الأطفال، فقد تولينا الاتصال بمؤلفي الدراسات. كما قمنا بتقييم عنصر عدم التجانس (Heterogeneity) وخطر الانحياز. ومن أجل الوقوف على مستوى نجاح العلاج – أي التوليفة ما بين إكمال العلاج وتحقيق الشفاء – فقد أجرينا تحليلاً تجميعيًا (Meta-analysis) للآثار العشوائية. وقمنا بالتالي بتحديد جوانب الاحتياج لتطوير إجراءات التدخل.

**النتائج:**

لقد ضمّنا 15 دراسة في 11 بلدًا حرصًا على التحليل النوعي لهذه النتائج، ومن بين هذه الدرسات فقد تأهلت 11 دراسة للتحليل التجميعي مثلت 1279 طفلاً. ومن بين التدخلات الموصوفة في الدراسات البالغ عددها 15 دراسة، ركزت اثنتان على التوعية، فيما انصب تركيز دراسة واحدة على الدعم النفسي والاجتماعي، واتجهت سبع دراسات إلى التركيز على تقديم الرعاية، فيما ركزت أربع دراسات على النظم الصحية، وانفردت دراسة واحدة بالتركيز على الاعتمادات المالية. وحقق الأطفال المسجلين في الفريق الخاضع لتجربة التدخل العلاجي نسبًا أعلى من نجاح العلاج مقارنةً بالأطفال المسجلين في المجموعات المرجعية (بنسبة احتمال بلغت 3.02؛ وبنسبة أرجحية مقدارها 95%: 2.19–4.15). وبالاعتماد على النتائج المستخلصة من تحليلاتنا، فقد وضعنا إطارًا سياقيًا يقوم على العوامل التي شجعت على اكتمال العلاج أو هددت اكتماله.

**الاستنتاج:**

يتبين أن التدخلات المختلفة الساعية إلى تحسين مستوى الالتزام بالعلاج الخاص بمرض السلّ لدى الأطفال في البلدان محدودة ومتوسطة الدخل كانت فعالة وذات جدوى. ****

## 摘要

**目的:**

针对在中低收入国家提高儿科结核病的治疗依从性，评估干预措施的设计、完成和效果，并为该种干预措施制定一个关联性框架。

**方法:**

我们在 PubMed 和 Cochrane 数据库中搜索了于 2003 年 1 月 1 日至 2013 年 12 月 1 日之间发表的报告，这些报告与提高结核病治疗依从性的干预措施有关，其中包括居住在中低收入国家且年龄不满 20 岁的患者。 相关文章可能缺少关于儿科的结果，为此，我们联系了研究的作者。 我们对异质性和偏倚风险进行了评估。 为了评估治疗成功率——即，完治率与治愈率相结合——我们采用随机效应开展了元分析。 我们确认了有必要对干预实践作出改善的领域。

**结果:**

我们将 11 个国家的 15 项研究纳入定向分析，其中 11 项符合元分析的条件——其代表 1279 名儿童。 关于 15 项研究中描述的干预措施，其中两项侧重于教育，一项侧重于社会心理支持，七项侧重于医疗保健服务，四项侧重于卫生体系，另外一项侧重于财政拨款。 与对照组的儿童相比，经干预措施庇佑的儿童具有更高的治疗成功率（比值比： 3.02；95% 置信区间： 2.19–4.15）。通过我们分析得出的结果，我们围绕促进或威胁完治率的因素制定了框架。

**结论:**

在中低收入国家为提高儿科结核病的治疗依从性而采取的多种干预措施显得可行、有效。

## Резюме

**Цель:**

Оценить структуру, реализацию и результаты медицинских вмешательств, способствующих соблюдению режима лечения детского туберкулеза в странах с низким и средним уровнем доходов населения, и разработать контекстуальную схему таких вмешательств.

**Методы:**

Был проведен поиск отчетов по медицинским вмешательствам, способствующим соблюдению режима лечения детского туберкулеза, опубликованным с 1 января 2003 г. по 1 декабря 2013 г. Поиск проводился в базах данных PubMed и Кокрановской библиотеки. Нас интересовали пациенты моложе 20 лет, проживающие в странах с низким или средним уровнем доходов. Если в потенциально релевантной статье было недостаточно данных по результатам лечения пациентов детского возраста, мы обращались за сведениями к авторам исследований. Была выполнена оценка гетерогенности данных и риска системной ошибки. Для оценки успешности лечения, т. е. завершения курса лечения и выздоровления пациента, был проведен метаанализ случайных воздействий. Были выявлены области, в которых практики медицинских вмешательств нуждаются в улучшении.

**Результаты:**

В количественный анализ было включено 15 исследований, выполненных в 11 странах. Из них 11 исследований было отобрано для метаанализа. В этих исследованиях было представлено 1279 детей. Среди описанных в них вмешательств две программы делали акцент на обучении, одна — на психологической поддержке, семь — на эффективности ухода, четыре касались систем здравоохранения и одна — предоставления финансирования. В тех группах, где проводилось вмешательство, дети имели более высокий показатель успешного лечения по сравнению с детьми из контрольных групп (отношение шансов: 3,02; 95% доверительный интервал: 2,19–4,15). Используя результаты наших анализов, мы разработали модель на базе факторов, которые способствуют или препятствуют завершению лечения.

**Вывод:**

Различного рода вмешательства, способствующие соблюдению режима лечения туберкулеза у детей, оказались осуществимой и эффективной стратегией для стран с низким и средним уровнем доходов населения.

## Introduction

Paediatric tuberculosis can be controlled or cured if timely and appropriate treatment is completed.^1^^,^^2^ More than 75% of affected patients live in low- and middle-income countries in Asia and Africa and have substantial tuberculosis –related morbidity and mortality.^2^ Up to 20% of children with tuberculosis in low- and middle-income countries fail to complete treatment.^3^

Interrupted tuberculosis treatment poses a public health challenge because it permits the development of drug-resistant disease and allows patients to remain infectious for a relatively long time. Poor adherence results in disease progression, morbidity and death. The most extreme form of incomplete treatment is known as treatment abandonment or treatment default. For tuberculosis, such abandonment is generally represented by a break in treatment of at least two consecutive months.^1^

The barriers to treatment completion in low- and middle-income countries include medical expenses, the indirect costs of transportation and time away from work, the stigmas associated with the illness and/or the treatment, communication breakdowns between providers and patients, limited health literacy, the presence of too few health workers and problems in drug procurement.^2^ We conducted a systematic review and meta-analysis of interventions designed to reduce such barriers to treatment completion among children with tuberculosis in low- and middle-income countries. Our main aim was to appraise the design, delivery and impact of such interventions in such a vulnerable population.

## Methods

### Search and selection

Using a registered protocol (PROSPERO: CRD42013005800), we searched the PubMed and Cochrane databases for relevant publications that had been published between 1 January 2003 and 1 December 2013. Grey literature was hand-searched. Until 1 May 2014, we attempted to contact the authors of relevant articles and other researchers with experience of tuberculosis in low- and middle-income countries. The search strategy (Box 1; available at: http://www.who.int/bulletin/volumes/93/10/14-147231) was piloted by two researchers and reviewed by two medical librarians.

Box 1 **Search strategy to identify studies on interventions to improve adherence to treatment for paediatric tuberculosis in low- and middle-income countries**

(“low income economies” OR “lower middle income economies” OR “middle income economies” OR “developing countries”[MeSH Terms] OR (“developing”[All Fields] AND “countries”[All Fields]) OR “developing countries”[All Fields]) OR (“developing countries”[MeSH Terms] OR (“developing”[All Fields] AND “countries”[All Fields]) OR “developing countries”[All Fields] OR (“developing”[All Fields] AND “country”[All Fields]) OR “developing country”[All Fields]) OR (“developing countries”[MeSH Terms] OR (“developing”[All Fields] AND “countries”[All Fields]) OR “developing countries”[All Fields] OR (“underdeveloped”[All Fields] AND “countries”[All Fields]) OR “underdeveloped countries”[All Fields]) OR (“developing countries”[MeSH Terms] OR (“developing”[All Fields] AND “countries”[All Fields]) OR “developing countries”[All Fields] OR (“underdeveloped”[All Fields] AND “country”[All Fields]) OR “underdeveloped country”[All Fields]) OR (emergent[All Fields] AND countries[All Fields]) OR (emergent[All Fields] AND country[All Fields]) OR (“developing countries”[MeSH Terms] OR (“developing”[All Fields] AND “countries”[All Fields]) OR “developing countries”[All Fields] OR (“developing”[All Fields] AND “nation”[All Fields]) OR “developing nation”[All Fields]) OR (underdeveloped[All Fields] AND “nation”[All Fields])) OR (emergent[All Fields] AND “nation”[All Fields]) OR ((“poverty”[MeSH Terms] OR “poverty”[All Fields] OR (“low”[All Fields] AND “income”[All Fields]) OR “low income”[All Fields]) AND countries[All Fields]) OR ((“poverty”[MeSH Terms] OR “poverty”[All Fields] OR (“low”[All Fields] AND “income”[All Fields]) OR “low income”[All Fields]) AND country[All Fields]) OR angola OR Fij OR palau OR albania OR gabon OR panama OR algeria OR grenada OR peru OR american samoa OR hungary OR romania OR argentina OR iran OR serbia OR azerbaijan OR iraq OR seychelles OR belarus OR jamaica OR south africa OR belize OR jordan OR st. lucia OR bosnia and herzegovina OR kazakhstan OR st. vincent and the grenadines OR botswana OR lebanon OR suriname OR brazil OR libya OR thailand OR bulgaria OR macedonia, fyr OR tonga OR china OR malaysia OR tunisia OR colombia OR maldives OR turkey OR costa rica OR marshall islands OR turkmenistan OR cuba OR mauritius OR tuvalu OR dominica OR mexico OR venezuela, rb OR dominican republic OR montenegro OR ecuador OR namibia OR armenia OR india OR samoa OR bhutan OR kiribati OR sao tome and principe OR bolivia OR kosovo OR senegal OR cameroon OR Lao OR solomon islands OR cape verde OR lesotho OR sri lanka OR congo OR mauritania OR sudan OR cote d'ivoire OR ivory coast OR micronesia OR swaziland OR djibouti OR moldova OR syria OR egypt OR mongolia OR timor OR el salvador OR morocco OR ukraine OR georgia OR nicaragua OR uzbekistan OR ghana OR nigeria OR vanuatu OR guatemala OR pakistan OR vietnam OR guyana OR papua new guinea OR west bank OR gaza OR honduras OR paraguay OR yemen OR indonesia OR philippines OR zambia OR afghanistan OR gambia OR myanmar OR bangladesh OR guinea OR nepal OR benin OR niger OR burkina faso OR haiti OR rwanda OR burundi OR kenya OR sierra leone OR cambodia OR korea OR somalia OR central african republic OR kyrgyz OR sudan OR chad OR liberia OR tajikistan OR comoros OR madagascar OR tanzania OR congo OR malawi OR togo OR eritrea OR mali OR uganda OR ethiopia OR mozambique OR zimbabwe)) AND tuberculosis[MeSH Major Topic] AND (“Health Education”[Mesh] OR “Counseling”[Mesh] OR “Directive Counseling”[Mesh] OR “Health Promotion”[Mesh] OR “Reminder Systems”[Mesh] OR “Directly Observed Therapy”[Mesh] OR “Social Support”[Mesh] OR “Contracts”[Mesh] OR “Decision Support Techniques”[Mesh] OR intervention OR treatment OR outcome) AND (study OR trial) AND (“Treatment Refusal”[Mesh] OR “Patient Participation”[Mesh] OR “Patient Dropouts”[Mesh] OR “Patient Compliance”[Mesh] OR “Motivation”[Mesh] OR “Cooperative Behavior”[Mesh]) OR “Refusal to Treat”[Mesh]) OR “Medication Adherence”[Mesh] OR medication adherence OR nonadherence OR non-adherence OR compliance OR noncompliance OR abandonment of treatment OR abandonment of therapy OR treatment abandonment OR therapy abandonment OR treatment default OR lost to follow-up OR loss to follow up OR default* OR against medical advice OR abscond* OR refusal OR stop* treatment OR (interrupt* AND treatment) OR (treatment AND discontinu*) OR (treatment AND continu*) OR failure to complete treatment OR incomplete treatment OR treatment maintenance OR no show OR retention of care OR run away OR attrition)) AND (“last 10 years”[PDat] AND Humans[Mesh] AND (infant[MeSH] OR child[MeSH] OR adolescent[MeSH] OR “young adult”[MeSH]) NOT “case reports”[Publication Type]) NOT “review”[Publication Type]

To be included in our analyses, a study had to have participants with active tuberculosis who were younger than 20 years and lived in a country that, according to the World Bank, was low-income or middle-income in December 2013. Studies with adult participants were included only if the cohort outcomes for participants younger than 20 years were available. We were only interested in studies on interventions targeted at the improvement of treatment initiation or completion, the improvement of adherence to medications or appointments, the prevention of treatment refusal or adherence surrogates such as self-efficacy or enablement.

Included studies required a control or comparison population. Retrospective or contemporaneous comparisons from the same region were accepted if the between-population similarities and differences were clearly stated. No language, follow-up or study quality restrictions were imposed.

### Data extraction

By using standardized forms, two investigators independently screened abstracts and extracted data. Discrepancies between the two investigators were resolved through discussion (16 records) or by the seeking of clarification from an author of an article of potential interest (three records).

We detected 62 studies that met all of our eligibility criteria apart from the provision of explicit outcomes for paediatric patients. Although we attempted to determine such outcomes by contacting the authors of the corresponding study reports, we successfully obtained outcomes for just 10 additional studies. The other 52 reports provided no current contact information for any author (14 studies), had authors who did not reply to our queries (20 studies) or had authors who stated that the data we wanted were not available (18 studies).

From each eligible report, we extracted information on methods, interventions, outcomes, participants, settings and co-infection with human immunodeficiency virus (HIV). Treatment outcomes were extracted according to the World Health Organization’s (WHO’s) classifications, with treatment success defined as completion or cure^1^ – as given in the reports.

Risk of bias in the randomized trials was assessed using the Cochrane Assessment tool^4^ and reported according to CONSORT standards.^5^ Quality of the non-randomized trials was assessed using the Effective Public Health Practice Project Quality Assessment tool^6^ and reported according to TREND standards.^5^^,^^7^ Funding source was recorded as a possible bias source. Studies that integrated qualitative data were assessed using the relevant tools of the Critical Appraisal Skills programme.^8^ Reporting of the systematic review adhered to the Preferred Reporting Items for Systematic Reviews and Meta-Analyses statement.^9^

Interventions to improve treatment adherence among paediatric patients of tuberculosis were summarized through independent iterative re-reading and organization of the identified themes – with discussion to achieve consensus – in alignment with WHO’s adherence dimensions for long-term therapies.^2^ For the initial data extraction, interventions were divided into five categories: education, psychosocial, care delivery, health systems and social protection or financial (Table 1). We attempted to determine those factors that promoted or threatened treatment completion. These factors might be related to: (i) the patient – e.g. literacy, (ii) the condition, including the presence of comorbidities, (iii) the therapy, including cultural lay beliefs, (iv) the health system, including accessibility, and (v) socioeconomic status, including family income.

**Table 1:** Categorization of interventions aimed at improving tuberculosis treatment adherence

Intervention category	Components	Examples
Education	Behavioural and cognitive	Teaching of patients, family members and community members
Psychosocial	Behavioural and affective	Counselling
		Contracts
		Cultural competence contextualization
		Social support to include communication relevant to patient efficacy or enablement
Care delivery	Behavioural, affective, biological and structural	Treatment regimen interventions in the form of combination pills or easier dosing
		Convenience of visits timed with medication refills
		Staff training – including provider-targeted interventions related to communication
		Decentralization of health contact via home visits or community health workers
Health systems	Behavioural, biological, cognitive and structural	Management processes
		Tracer systems
		Referral support
		Direct accountability in the form of direct observation of therapy
Social protection or financial	Behavioural and structural	Financial support for – or provision of – food, transportation and housing
		Free health services or reimbursement of costs

### Statistical analysis

We did a meta-analysis of the treatment success rates recorded among paediatric patients. We used the Mantel-Haenszel model and the DerSimonian and Laird random-effects method to calculate odds ratios (ORs) and their 95% confidence intervals (CIs) from the unadjusted raw data, with the assumption that intervention effects on treatment success in one setting might differ from those in other settings. We did sensitivity analyses that included only randomized or quasi-randomized studies or excluded studies with comparison population estimates derived from another setting (available from the corresponding author). Heterogeneity across studies was assessed using the *I^2^* statistic. We summarized the main meta-analysis results as a forest plot but used funnel plots to assess publication bias. Analyses were conducted using Review Manager version 5.2 (Cochrane Collaboration, Copenhagen, Denmark).

## Results

**Fig. 1:**
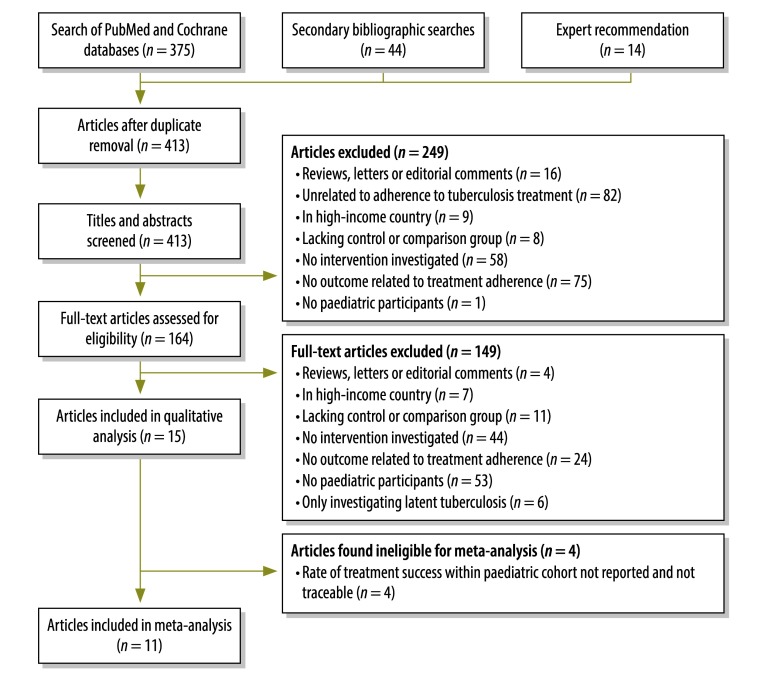
Flowchart for the selection of studies on interventions to improve adherence to treatment for paediatric tuberculosis in low- and middle-income countries

We initially identified 413 articles of potential interest. Of these, 164 qualified for full-text review and we included 15 articles in our qualitative synthesis (Fig. 1).^10^^–^^24^ The articles were on 15 separate studies (Table 2). Three of the studies were published in Portuguese^11^^,^^13^^,^^14^ and the remainder in English. Five studies were based in the upper-middle-income countries of Brazil^11^^,^^13^^,^^14^ and Thailand,^15^^,^^16^ three in the lower-middle-income countries of India,^12^ Lesotho^10^ and Pakistan,^17^ and seven in the low-income countries of Bangladesh,^23^ Ethiopia,^18^^,^^19^ Kenya,^22^ Myanmar,^24^ South Sudan^21^ and the United Republic of Tanzania.^20^ Four settings were urban outpatient,^11^^,^^13^^,^^16^^,^^17^ three rural outpatient,^12^^,^^18^^,^^19^ two suburban outpatient,^23^^,^^24^ one rural camp.^21^ The remaining studies were done in variable settings.^10^^,^^14^^,^^15^^,^^20^^,^^22^

**Table 2:** Studies on interventions to improve treatment adherence for paediatric tuberculosis in low- and middle-income countries, 1996–2011

Study	Country and study design	Care setting	Participant description	Duration, months	Period	Study arms	
						Intervention	Comparison
**Non-randomized**							
Anuwatnonthakate et al.^15^	Thailand, prospective observational cohort^a^	Region – all public and private facilities in four provinces	Diverse patient population including urban, rural and migrant populations. HIV co-infection rate 20%. Of the participants, 223 (3%) were aged < 15 years^b^.	24	2004–2006	DOT supervised by family member or HCW	Self-administered therapy
Heck et al.^11^	Brazil, retrospective observational cross-sectional^a^	City – 18 urban outpatient primary health units and five referral units supervised by Municipal Tuberculosis Control Programme	Socioeconomic and education summary not provided; HIV co-infection rate 16%. Of the participants, 57 (9%) were aged ≤ 19 years.	96	2000–2004 and 2005–2008	Decentralization of tuberculosis programme actions for primary care and implementation of DOT	SOC before decentralization initiatives
Lee et al.^23^	Bangladesh, prospective before-and-after study^a^	Clinic – suburban primary health clinic in industrial complex near capital	Participants had low socioeconomic status, limited education and high level of illiteracy. Of the participants, 26 (7%) were aged < 18 years^b^.	33	2005–2006 and 2006–2007	Patient education on the importance of treatment adherence provided, by a physician, weekly for 1 month, fortnightly for next month, then monthly. Visits scheduled to coincide with medication refills	SOC, with no standardized patient education and return visits not timed to coincide with refills
Marques and da Cunha^14^	Brazil, retrospective before-and-after^a^	Hospital – urban hospital	Indigenous population suffering extreme poverty, malnutrition and cultural and socioeconomic barriers to extended hospitalization. Of the participants, 244 (41%) were aged < 15 years^b^.	35	1996–1998 and 1998–1999	Outpatient treatment with home-based DOT via indigenous health agents	Systematic hospitalization of patients for up to 6 months
Ong’ang’o et al.^22^	Kenya, retrospective cohort^a^	Region – sample of four urban and rural public health facilities, using and not using CHWs	Mention of stigma towards tuberculosis and cultural beliefs against conventional treatment of the disease in rural setting. Of the participants, 298 (11%) were aged < 14 years^b^.	72	2005–2011	Personalized education from CHW, on treatment and risks involved in lack of adherence, plus CHW-supervised DOT at household level with ongoing CHW educational support	Nurse at health facility advised patients of treatment schedule, need for adherence and need for family support. Weekly DOT at health facility
Satti et al.^10^	Lesotho, retrospective cohort	Community – mountainous rural and urban, inpatient and outpatient setting	Nineteen patients with suspected or confirmed MDR tuberculosis, of whom 14 (74%) were co-infected with HIV, 12 (63%) were malnourished and all were aged < 16 years.	42	2007–2011	Comprehensive approach to care for MDR tuberculosis, with or without HIV co-infection, using social support, close monitoring by CHWs and clinicians and inpatient care when warranted	Patients of MDR tuberculosis with high rates of HIV co-infection in neighbouring South Africa
van den Boogaard et al.^20^	United Republic of Tanzania, retrospective observational cohort^a^	Region – urban and rural districts with national referral hospital, regional hospital and primary health clinics	Socioeconomic and education summary not provided. HIV co-infection rate 31%. Of the participants, 308 (11%) were aged < 15 years.	12	2007	Patient-centred treatment that allowed patients to choose between community and facility-based DOT	Conventional facility-based DOT supervised by facility-based provider
Badar et al.^17^	Pakistan, prospective observational cohort	Province – urban, nongovernment outpatient tertiary care hospital as referring centre	Socioeconomic and education summary not provided. Of the participants, 150 (34%) were aged ≤ 19 years.	9	2009	Electronic database register, designated oversight of referrals, staff referral orientation, tracking via 1–3 phone calls, communication between centres via exchanges of pre-stamped mail, scheduled meetings and phone contact and patients referred to closest facility	Patient responsible for return to referring unit
Cantalice Filho^13^	Brazil, before-and-after^a^	Clinic – urban primary health care outpatient clinic	Socioeconomic and education summary not provided. HIV co-infection rate < 5%. Of the participants, 8 (6%) were aged < 18 years^b^.	57	2001–2003 and 2004–2006	Standard treatment regimen plus monthly food basket	Standard treatment regimen, including self-administered therapy
Keus et al.^21^	South Sudan, prospective observational cohort^a^	Programme – humanitarian rural tuberculosis camp located in “transitional” zone between militia and local factions	Pastoral, migratory population living in conflict conditions with no health infrastructure. HIV co-infection rate < 5%. Of the participants, 84 (52%) were aged < 15 years.	9	2001	Village-based treatment in a conflict zone of South Sudan	Treatment in a less insecure area – Manyatta Region – with 2-month supervised then 3-month unsupervised regimen
Lönnroth et al.^24^	Myanmar, prospective cohort	Clinics – multiple township outpatient clinics serving low-income population	Mostly patients with low socioeconomic status, from townships in which many used private health care as the first point of contact. Of the participants, 66 (26%) were aged 16 years.	14	2004–2005	Social franchise engaging private general practitioners to deliver quality controlled tuberculosis care, including service branding, defined treatment supporter and default tracing mechanism	Continuation of previous SOC, with patient utilization of existing treatment centres and the public sector’s DOT logo branding
**Randomized or quasi-randomized**							
Datiko and Lindtjørn^18^	Ethiopia, prospective randomized	Clinics – rural outpatient setting in south of country	Patients with poor access, poverty and low health-seeking behaviours. Of the participants, 32 (10%) were aged < 14 years.	19	2006–2008	Local treatment by HEWs. HEW training in adherence support, diagnosis, referral with enhanced case finding and the problems of non-adherence. Community mobilization and education	HEWs did not receive training on diagnostic techniques or adherence support. HEWs engage in community education on symptoms of tuberculosis. DOT provided at health facility instead of within local neighbourhood
Demissie et al.^19^	Ethiopia, prospective quasi-randomized^a^	Clinics – rural outpatient centres in north of country	Tuberculosis associated with strong community stigma, to the extent that patients may lose their work if employer is aware of diagnosis. Of the participants, 7 (5%) were aged < 15 years.	12	1998–1999	Patients organized according to residential area into clubs, each with 3–10 members, an elected leader and the same appointment dates. Weekly club meetings with emphasis on social support towards treatment completion.	Continuation of previous SOC. No tuberculosis clubs but otherwise similar treatment regimen and packages of health education as in the intervention arm
Khortwong and Kaewkungwal^16^	Thailand, prospective quasi-randomized^a^	Clinics – urban outpatient hospital clinics	Marginalized migrant population living in crowded conditions, with high mobility. Lack of legal status or registration made most ineligible for routine health-care services. Of the participants, 4 (4%) were aged < 18 years^b^.	16	2009–2010	Migrant population provided with intensive education modules, home and workplace visits and phone-call reminders, with emphasis on therapeutic health team relationships	Migrant population received continuation of previous SOC, which included optional treatment supervision by a village health volunteer
Mathew et al.^12^	India, retrospective quasi-randomized observational cohort	Clinic – outpatient clinic based in rural secondary-level mission hospital in north of country	In one of the poorest regions in India, with high rate of illiteracy. Tribal population engaged in small-scale farming, with poor road access. Of the participants, 94 (14%) were aged < 15 years but data were only reported for 61 of these.	30	2001–2003	Free drugs, visits made to the patient by the DOT supervisor – a community member – monthly during intensive phase and every 2 months thereafter. Adherence checks. Patient asked to visit clinic three times during therapy	Drugs provided at cost, family member supported DOT and accompanied patient to appointments. Monthly clinic visits in intensive phase and clinic visits every 2 months thereafter

CHW: community health worker; DOT: directly observed therapy; HCW: health-care worker; HEW: health-extension worker; HIV: human immunodeficiency virus; MDR: multidrug-resistant; SOC: standard of care.

^a^ An author of the relevant article had to be contacted to clarify the rate of treatment success in the paediatric participants and/or the definition used for treatment abandonment.

^b^ The size of the paediatric sample has not been published previously and had to be obtained by direct contact with an author of the relevant article.

The payment system for health services was not described in nine studies^11^^,^^12^^,^^14^^–^^20^ but the reports on four studies described capped fees^24^ or clinic fee coverage.^16^^,^^23^^,^^24^ In seven studies, drug expenses were covered for one intervention group only,^12^ for both the intervention and comparison groups, as part of a national scheme,^16^^,^^22^^–^^24^ or for at least the intervention group – with unclear indication if the drug expenses of the comparison group were also covered.^10^^,^^21^

The included studies were conducted between 1996 and 2011 and reported – including the unpublished data supplied by authors – between 2003 and 2014. The median duration of the investigated interventions was 24 months (range: 9–96). The number of participants younger than 20 years – which had to be clarified through author contact for six studies and excluded population-based comparison samples – varied from four to 308 (mean: 106; median: 61) and totalled 1587 across all 15 studies. Such paediatric patients represented between 3% and 100% of the patients investigated (mean: 22%; median: 11%). The prevalence of HIV co-infection, which was only reported for six studies, ranged from less than 5% to 74%.^10^^,^^11^^,^^13^^,^^15^^,^^20^^,^^21^

### Interventions

The timing of interventions either included referral^10^ or induction^15^ or ran just from treatment initiation to treatment completion.^11^^–^^14^^,^^16^^–^^24^ Health behaviour models informing intervention design were mentioned in two studies – the precede-proceed model was used to help engage patients in one study^16^ while social franchising was used to help engage providers in another study.^24^

Many studies involved several categories and subcategories of interventions (Table 3). Some used interventions combining cognitive and behavioural components, as exemplified by education for patients,^10^^,^^12^^,^^16^^,^^18^^,^^19^^,^^21^^–^^24^ family members,^10^^,^^12^^,^^20^^,^^21^ or community leaders.^12^^,^^18^^,^^19^^,^^21^^,^^24^ Educational curricula addressed the administration^11^^,^^16^^,^^18^^–^^20^^,^^22^^,^^23^ and adverse effects of medication,^16^^,^^19^^,^^23^^,^^24^ the personal or public health consequences of early treatment discontinuation^16^^,^^19^^,^^21^^–^^23^ and overall health or hygiene.^16^^,^^18^^,^^19^^,^^21^

**Table 3:** Interventions to improve adherence to treatment for paediatric tuberculosis in low- and middle-income countries, 1996–2011

Main category of primary intervention, reference	Intervention categories and subcategories included in study																						
	Educational				Psychosocial						Care delivery					Health systems			Social protection or financial				
	Provider	Patient	Family	Community	Therapeutic alliance^a^	Peer support	Counselling	Stigma addressed	Staff support	Patient-centred choices	Scheduling	Decentralization	Staff training	Care quality assurance	Treatment convenience	Directly observed treatment	Registry	Tracing	Food	Transport	Living environment	Income generation	Subsidized treatment
**Educational**																							
Khortwong and Kaewkungwal^16^	–	+	–	–	+	+	+	–	+	+	–	+	+	–	–	+	–	+	+	–	+	–	–
Lee et al.^23^	–	+	–	–	+	–	–	–	+	–	+	–	–	–	–	–	–	–	–	–	–	–	–
**Psychosocial**																							
Demissie et al.^19^	–	+	–	+	+	+	–	+	+	–	+	–	+	–	–	+	–	+	–	–	–	–	–
**Care delivery**																							
Anuwatnonthakate et al.^15^	–	–	–	–	–	–	–	–	+	+	–	+	–	–	–	+	–	–	–	–	–	–	–
Datiko and Lindtjørn^18^	–	+	–	+	+	–	–	–	–	+	–	+	+	+	+	+	–	+	–	–	–	–	–
Heck et al.^11^	–	–	–	–	–	–	–	–	–	–	–	+	–	–	–	+	–	–	–	–	–	–	–
Keus et al.^21^	+	+	+	+	+	+	+	+	+	–	–	+	+	+	+	+	–	+	+	–	+	–	–
Marques and da Cunha^14^	–	–	–	–	–	+	–	+	–	–	–	+	–	–	–	–	–	–	–	–	–	–	–
Satti et al.^10^	–	+	+	–	+	–	+	–	+	–	+	+	+	–	+	+	–	+	+	+	+	+	–
van den Boogaard et al.^20^	+	–	+	–	–	–	–	–	+	+	–	+	–	–	–	+	–	–	–	–	–	–	–
**Health systems**																							
Badar et al.^17^	–	–	–	–	–	–	–	–	–	–	–	–	+	–	–	–	+	+	–	–	–	–	–
Lönnroth et al.^24^	+	+	–	+	–	–	–	–	–	–	–	–	+	+	+	–	+	+	–	–	–	–	+
Mathew et al.^12^	–	+	+	+	–	–	–	–	+	+	+	+	–	+	+	+	–	+	–	–	–	–	+
Ong’ang’o et al.^22^	–	+	–	–	+	+	+	–	+	+	–	+	+	–	–	+	–	+	–	–	–	–	–
**Social protection or financial**																							
Cantalice Filho^13^	–	–	–	–	–	–	–	–	–	–	–	–	–	–	–	–	–	–	+	–	–	–	–

^a^ Refers to relationship-building between providers and patients**.**

Eleven studies incorporated affective and behavioural components, through psychosocial support with therapeutic alliances (i.e. relationship-building between providers and patients),^10^^,^^16^^,^^18^^,^^19^^,^^21^^–^^23^ patient empowerment to select a treatment supporter or location,^12^^,^^15^^,^^16^^,^^18^^,^^20^^,^^22^ counselling,^10^^,^^16^^,^^21^^,^^22^ problem-solving,^16^ decreasing stigma^14^^,^^19^^,^^21^ and peer support.^14^^,^^16^^,^^19^^,^^21^^,^^22^

Care delivery interventions included health provider training,^10^^,^^16^^–^^19^^,^^21^^,^^22^^,^^24^ convenient appointment scheduling,^10^^,^^12^^,^^19^^,^^23^ migration-sensitive therapy duration^21^ and easier dosing schedules.^10^^,^^12^^,^^18^^,^^24^ Health system interventions included the directly observed treatment, short-course strategy,^10^^–^^12^^,^^15^^,^^16^^,^^18^^–^^22^ referral support,^17^^,^^19^ patient tracers^10^^,^^12^^,^^16^^–^^19^^,^^21^^,^^22^^,^^24^ – including tracing within 24 hours^12^^,^^21^ – and home visiting.^16^^,^^22^

Social protection or financial support interventions included weekly food rations,^10^^,^^21^ monthly food baskets,^13^ housing,^21^ medication coverage,^12^^,^^21^ recognition of the importance of employment^14^^,^^16^ or school,^10^ essential supplies for daily life,^16^ transport reimbursement^10^ and income-generation support.^10^ One study required a deposit that was refundable upon treatment completion.^12^

### Treatment adherence

Adherence-related measures included those extracted from self-reports,^16^^,^^24^ pharmacy refill data,^23^ medication records maintained by treatment supporters,^12^^,^^19^ clinic attendance records,^23^ confirmation of referrals^17^ and medical records.^10^^,^^11^^,^^13^^–^^15^^,^^18^^,^^20^^–^^22^^,^^24^

Terminology describing unfavourable outcomes included default,^10^^,^^12^^,^^15^^,^^16^^,^^18^^–^^22^^,^^24^ drop-out,^11^^,^^14^ abandonment^13^^,^^14^ and treatment interruption.^19^ Three of 10 studies used the term default and, in defining their default criteria, were consistent with WHO definitions.^10^^,^^19^^,^^24^ Drop-out was defined in one study as treatment interruption for more than 30 days.^11^ Treatment abandonment was not defined in the two studies using the term.^13^^,^^14^

In addition to treatment success – i.e. completion or cure – positive outcomes were defined in the study reports as successful referral – i.e. confirmed arrival at the referral facility,^17^ continuous attendance at scheduled visits,^22^^,^^23^ more than 90% medication adherence^23^ or self-reported beneficial health behaviours.^16^

### Risk of bias

The benefits of the investigated interventions may be overestimated because of short follow-up and failure to assess adherence after the interventions were discontinued. Confounders, such as the extra attention given to participants during educational interventions,^16^^,^^23^ complicate our analyses. Although one study report details how controls – who did not receive the educational intervention – were supervised by health volunteers,^16^ it failed to give any idea of the corresponding contact time. The concurrent use of several interventions makes it hard to determine the main reason for successful outcomes. Social feedback loops – in which successful interventions foster a dynamic for more community adherence – were subjectively recognized by several research teams.^16^^,^^18^^,^^19^^,^^21^^,^^24^ Intervention complexity increased as attention expanded beyond the patient to include the provider,^23^ the family,^13^^–^^15^ both the provider and family^10^^–^^12^^,^^16^^,^^17^^,^^20^ or the provider, family and community.^18^^,^^19^^,^^21^^,^^22^^,^^24^ Complexity was characterized by contextual interactions that were susceptible to policy timing,^13^^,^^18^^,^^20^^,^^21^^,^^24^ staffing capabilities and attitudes,^12^^,^^16^^,^^17^^,^^19^^,^^22^^,^^23^ relationships^13^^,^^16^^,^^19^^,^^23^ and resources.^18^^,^^19^^,^^23^^,^^24^ No empiric quality measures of implementation fidelity were described.

Two studies incorporated qualitative data from focus groups and in-depth interviews.^19^^,^^22^ Although context, sampling and data collection were outlined and the findings appeared supported by data, there was no discussion of reflexivity and no detailed description of the analyses. None of the studies we investigated incorporated long-term observational or ethnographic approaches.

In one prospective randomized controlled trial, the study communities were randomly allocated to intervention and control groups to limit selection bias.^18^ Three quasi-randomized trials determined assignment by residence.^12^^,^^16^^,^^19^ No before-and-after studies used controls to account for any secular change. None of the articles described blinding measures and three specified a lack of blinding for assessors^11^^,^^24^ or participants.^20^

All of the results reported in thirteen studies were apparently defined a priori.^10^^–^^16^^,^^18^^–^^20^^,^^22^^–^^24^ The remaining two studies accounted for modification of the results reported due to limited follow-up data, which had impaired the assessment of cure^21^ or treatment outcome beyond referrals.^17^

Funding sources included nongovernmental organizations,^10^^,^^11^^,^^20^^–^^24^ health departments^18^ or international^15^^,^^17^^,^^19^ or local^16^ academic institutes or were not specified.^11^

Table 4 and Table 5 show the results on study-specific biases (available at: http://www.who.int/bulletin/volumes/93/10/14-147231).

**Table 4:** Assessment of non-randomized studies on interventions to improve adherence to treatment for paediatric tuberculosis in low- and middle-income countries

Study	Selection bias	Study design	Confounders	Blinding	Data collection method	Withdrawals and dropouts	Global rating
Anuwatnonthakate et al.^15^	Moderate	Moderate	Strong	Weak	Weak	Strong	Weak
Heck et al.^11^	Moderate	Weak	Weak	Weak	Weak	Moderate	Weak
Lee et al.^23^	Moderate	Moderate	Strong	Not clear	Weak	Moderate	Moderate
Marques and da Cunha^14^	Not clear	Moderate	Weak	Not clear	Weak	Weak	Weak
Ong’ang’o et al.^22^	Moderate	Moderate	Strong	Moderate	Weak	Strong	Moderate
Satti et al.^10^	Moderate	Weak	Weak	Not clear	Weak	Strong	Weak
van den Boogaard et al.^20^	Moderate	Moderate	Moderate	Weak	Weak	Moderate	Weak
Badar et al.^17^	Not clear	Weak	Weak	Weak	Weak	Weak	Weak
Cantalice Filho^13^	Moderate	Moderate	Moderate	Not clear	Weak	Weak	Weak
Keus et al.^21^	Moderate	Weak	Weak	Moderate	Weak	Strong	Weak
Lönnroth et al.^24^	Weak	Weak	Weak	Not clear	Weak	Strong	Weak

Note: Assessed by using Effective Public Health Practice Project Quality Assessment Tool for Quantitative Studies.

**Table 5:** Risk of bias in randomized control and quasi-randomized control studies on interventions to improve adherence to treatment for paediatric tuberculosis in low- and middle-income countries

Study	Random sequence generation	Allocation concealment	Blinding of participants and personnel	Blinding of outcome assessors	Incomplete outcome data	Selective reporting	Other bias
Datiko and Lindtjørn^18^	Low	High	Low	High	Low	Low	Low
Demissie et al.^19^	High	Unclear	Unclear	High	Low	Unclear	Low
Khortwong and Kaewkungwal^16^	High	Unclear	Unclear	High	Low	Unclear	Low
Mathew et al.^12^	High	Unclear	High	High	High	Unclear	Low

Note: Assessed by using Cochrane criteria for judging risk of bias.^25^

### Meta-analysis

Treatment success rates for the paediatric participants in both the treatment and comparison groups were reported for 11 studies.^10^^–^^12^^,^^14^^–^^16^^,^^18^^–^^20^^,^^22^^,^^23^ These studies were included in the meta-analysis and together represented 1279 children – excluding those in any external comparison groups. In three of the four studies excluded from the meta-analysis, the interventions investigated appeared to bring improved rates of treatment success, for all age groups.^13^^,^^21^^,^^24^ The results of the other excluded study^17^ indicated that the intervention led to increased referral rates.

**Fig. 2:**
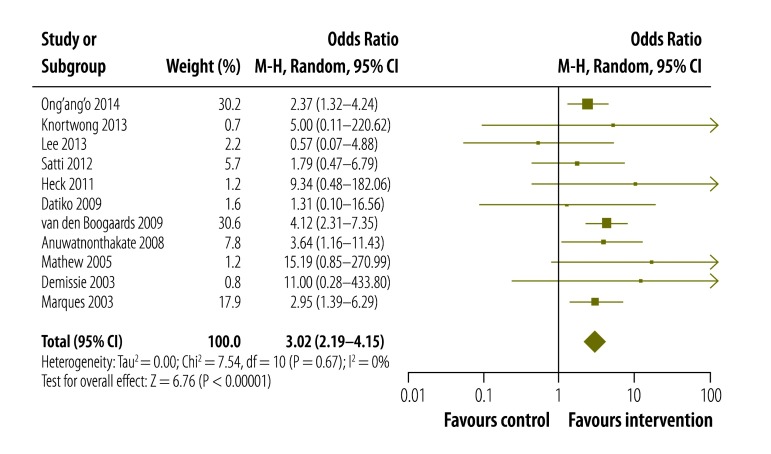
Effect on the odds of treatment success of interventions to improve adherence to treatment for paediatric tuberculosis in low- and middle-income countries, 1996–2011

CI: confidence interval; M-H: Mantel-Haenszel model.

Note: In a random effects meta-analysis, odds ratios were derived from individual studies (squares) or as summary value (diamond). The size of the square data marker for individual studies is proportional to the number of patients in the study.

**Fig. 3:**
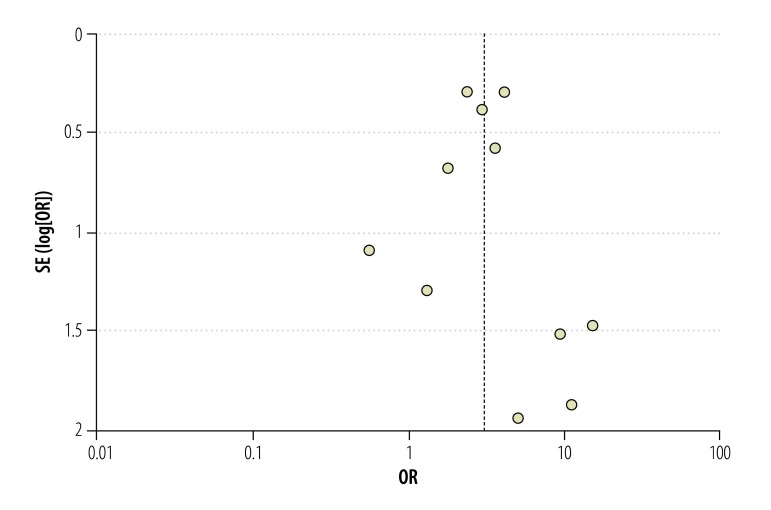
Funnel plot to evaluate publication bias of studies on interventions to improve adherence to treatment for paediatric tuberculosis in low- and middle-income countries

OR: odds ratio; SE: standard error.

Note: The dashed line represents the summary odds ratio derived from the meta-analysis. Odds ratios have been plotted on a logarithmic scale.

Meta-analysis revealed a threefold improvement in odds of treatment success for children receiving the interventions (Fig. 2; OR: 3.02; 95% CI: 2.19–4.15). There was no evidence of statistical heterogeneity (*I^2^*: 0%). A funnel plot showed symmetry for the large, high-powered studies but potential publication bias for the smaller studies (Fig. 3; available at: http://www.who.int/bulletin/volumes/93/09/14-147231). Sensitivity analysis did not modify the overall results (available from the corresponding author). Baseline risk factors reported for poor adherence outcomes are outlined in Box 2.

Box 2 **Reported risk factors for poor tuberculosis treatment adherence outcomes in paediatric patients**

**Patient-related**

- Female sex^12^
- Male sex^14^^,^^23^

**Condition-related**

- Human immunodeficiency virus-positive_20_
- Smear-negative tuberculosis^20^^,^^23^

**Treatment-related**

- Tuberculosis retreatment^24^

**Social and/or economic related**

- Low-socioeconomic level^24^

**Health system related**

- Distance from care source^12^

## Discussion

In our review of interventions to promote paediatric tuberculosis treatment adherence in low- and middle-income countries, we found evidence that such interventions can result in clinically important improvements in tuberculosis treatment success. Diverse interventions addressing education, psychosocial support, care delivery, health system strengthening and social protection are reportedly feasible and effective in facilitating treatment completion.

Several studies followed collaborative strategies. For example, there was evidence of social franchise programmes communicating with the media, tuberculosis villages communicating with local leaders, tuberculosis clubs communicating with neighbours, health centres communicating with referral facilities and health providers engaging in motivational communication with patients.

We used systematic methods to identify and analyse a broad range of studies, without language limitations and with solicitation of input from the authors of relevant articles in an attempt to minimize search bias. We provided detailed descriptions and syntheses of interventions – which were often multi-component and complex – that had been implemented among children in low- and middle-income countries. Our summary findings may help guide future intervention planning and evaluation. Our reviews did, however, have several limitations. For example, few studies included specific details on the nature of their paediatric programme, and no data on individual patients were available. Given the generally small sample sizes, the reported confidence intervals for the effects of individual interventions were often broad. Despite this, all but one of the 11 studies included in the meta-analysis had odds ratios that indicated that the investigated intervention improved the rate of treatment success, and the four largest of these studies provided unequivocal evidence of such benefit.

Heterogeneity in the context and measurement of adherence, outcome definition and reporting limit the value of between-study comparisons. In high-income countries, multi-component interventions are common and often found to be superior to single-component interventions.^26^ Several of the relevant studies included in our reviews also attempted to target several adherence factors simultaneously, by using complex interventions. Such complex interventions make it difficult to attribute the results to particular intervention categories or components. One of the studies we reviewed was of an intervention that included education, improved dosing and appointment convenience, patient tracing, reduction of out-of-pocket costs and a deposit that was refunded on treatment completion.^12^ It may be that only when implemented together do these elements succeed.

**Fig. 4:**
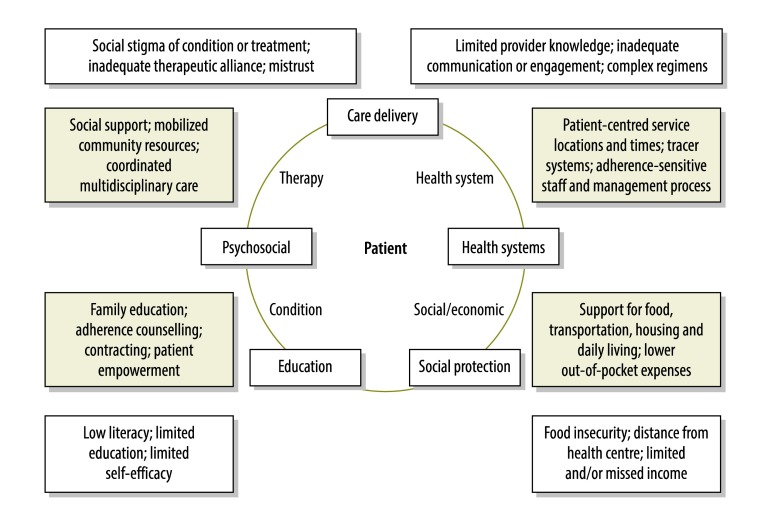
Contextual framework showing factors that may promote or threaten adherence to treatment for paediatric tuberculosis in low- and middle-income countries

Notes: The central circle, which contains the adherence dimensions used by the World Health Organization, is surrounded by the five main categories of relevant interventions. The factors that may promote treatment adherence are shown in green boxes and factors that may threaten treatment adherence are shown in white boxes. Therapeutic alliance refers to****relationship-building between providers and patients**.**

Recognizing the interconnected nature of WHO’s five adherence dimensions and intervention categories for long-term therapies,^2^ we have summarized contextual factors affecting the adherence interventions we investigated in a framework (Fig. 4). The themes highlighted in this figure are intended to be illustrative across dimensions and intervention categories. For instance, factors that may adversely affect tuberculosis treatment adherence that span psychosocial and educational categories – e.g. low literacy and limited self-efficacy – are shown in the figure alongside adherence-promoting factors such as family education and patient empowerment. The contextual framework may aid further collaborative studies and analyses of adherence-targeted interventions.

Through qualitative analysis, we identified three areas where studies described – or failed to describe – children’s unique features that can affect adherence intervention delivery. First, few studies described paediatric-specific disease epidemiology and use of paediatric-inclusive outcomes. Several authors reported an unexpectedly high prevalence of paediatric tuberculosis that warranted management as a public health problem.^14^^,^^18^^,^^21^However, most of the studies that we screened simply excluded children and 54 studies that would otherwise have been eligible for our analyses had to be excluded because they failed to report paediatric outcomes separately. Even for the eligible studies, adherence outcomes were not explicitly adapted for paediatric patients – although paediatric-specific treatment toxicity was recognized in one study.^10^

Second, several reports noted challenges in paediatric tuberculosis diagnosis and care. Children can pose diagnostic dilemmas that complicate epidemiological and outcome estimates.^10^^,^^21^ One study noted that paediatric lymph-node biopsies could not be safely performed locally.^21^ Another considered how children’s difficulty with sputum production may contribute to low detection rates^18^ while a different study specified distinct sputum collection techniques for younger children.^10^ Dosing instructions that were adapted for paediatric treatment were also recommended.^10^ Key comorbidities in children – e.g. malnutrition^21^ – may benefit from dedicated attention.

Third, several studies acknowledged the need to consider the preferences and social role of children and adolescents, who may need tailored interventions. In one study involving the use of directly observed, short-term treatment, children and women were more likely than men to select community-based over facility-based treatment, when given the option.^20^ Another study adapted an intervention, for use among children, according to household and social needs. This intervention included supporting the children in returning to school.^10^ As one study commented, tuberculosis – and tuberculosis treatment – can cut the economic productivity of adolescents and young adults, who tend to have relatively high burdens of the disease.^12^

Based on our review and identified themes, future studies need to: (i) assess interventions in low- and middle-income countries that explicitly analyse paediatric-inclusive and paediatric-distinct needs and outcomes, (ii) use mixed-method approaches that can assess the pathways linking context-dependent factors with outcomes, (iii) use longitudinal evaluations that investigate the sustainability of the effectiveness and benefits of interventions and the potential burdens posed by interventions, and (iv) incorporate and address cost–effectiveness, resource implications and potential scalability.

Our findings indicate the potential usefulness of diverse interventions to increase the rate of treatment completion among paediatric tuberculosis patients and improve outcomes in resource-poor settings.
